# Editorial: Natural compounds in veterinary therapeutics, volume II

**DOI:** 10.3389/fvets.2026.1943862

**Published:** 2026-09-02

**Authors:** Baocheng Hao, Dongan Cui, Shuaiyu Wang

**Affiliations:** 1 Key Laboratory of New Animal Drug Project, Lanzhou, Gansu, China; 2 Key Laboratory of Veterinary Pharmaceutical Development, Ministry of Agriculture and Rural Affairs, Lanzhou, China; 3 Lanzhou Institute of Husbandry and Pharmaceutical Sciences of Chinese Academy of Agriculture Sciences, Lanzhou, China; 4 College of Veterinary Medicine, Lanzhou University, Lanzhou, China; 5 College of Veterinary Medicine, China Agricultural University, Beijing, China

**Keywords:** immunomodulation, intestinal health, natural compounds, pharmacologic mechanism, safety evaluations, veterinary therapeutics

This editorial summarizes the Research Topic “*Natural compounds in veterinary therapeutics, volume II*.” Accumulating evidence demonstrates that natural bioactive compounds from animals, plants, microorganisms, and their metabolites exert diverse pharmacological functions and hold great promise for animal disease therapy (1). Such natural-origin agents commonly feature multi-target, multi-pathway, and multi-mode bioactivities (2). Collectively, the published papers on this topic cover the chemical profiling of natural-product formulations, as well as their anti-inflammatory, immunomodulatory, antioxidant, growth-promoting, and gut–microbiota-regulating properties.

In terms of chemical characterization of herbal preparations, Duller et al. profiled components of Neurexan, a formulation that mitigates stress responses in domestic animals. Identified constituents included caffeine, vitexin, isovitexin, orientin, isoorientin, apigenin, and the alkaloid trigonelline. These chemical data lay a foundation for future target-oriented pharmacological investigation (Duller et al.).

Regarding the anti-inflammatory actions of natural bioactive molecules, one study evaluated chlorogenic acid (CGA) against cisplatin-induced acute kidney injury (AKI) in mice. Preventive CGA supplementation alleviates renal injury by enhancing antioxidant capacity, suppressing inflammatory cascades, and preserving renal tissue structure. This study provides key experimental evidence supporting CGA as a candidate for AKI prophylaxis (Zheng et al.).

Multiple submissions addressed intestinal health and mucosal immunity in production animals. In weaned piglets, combining short-chain fructooligosaccharides (scFOS), essential oils (EOs), and sodium humate improved intestinal maturation, mucosal immunity, and the gut microbiota in the early post-weaning phase, stabilizing gastrointestinal homeostasis during this vulnerable developmental window (Decundo et al.). Dandelion polysaccharides (TKP-L) improved intestinal barrier function and growth performance in lambs, likely by reshaping the ^*^Prevotella^*^ community and modulating arginine–succinate metabolism to boost host growth and immunity (Aersilan et al.). Dietary valonea tannins (300 mg/kg) improved broiler body weight, accompanied by a reprogrammed hepatic transcriptome and cecal microbiota.

Natural-drug active ingredients are shifting from empirical applications toward scientific, precise, and industrial-scale exploitation. In animal disease control, they serve as promising alternatives to antibiotics and underpin green animal husbandry, food–animal product safety, and public health protection. Deeper mechanistic research and industrial translation will further expand their roles in modern animal health management (3, 4).
